# Characteristics of velopharyngeal dysfunction in 22q11.2 deletion syndrome: a retrospective case-control study

**DOI:** 10.1186/s40463-020-00451-4

**Published:** 2020-07-31

**Authors:** Sebastiano Failla, Peng You, Chandheeb Rajakumar, Anne Dworschak-Stokan, Philip C. Doyle, Murad Husein

**Affiliations:** 1 Voice Production and Perception Laboratory, Rehabilitation Sciences, Western University, London, Ontario Canada; 2 Department of Otolaryngology-Head and Neck Surgery, Children’s Hospital, London Health Sciences Center, Western University – Schulich School of Medicine, Victoria Campus, 800 Commissioners Road East, London, ON N6A 5W9 Canada; 3 Thames Valley Children’s Centre, London, Ontario Canada; 4 Voice Production and Perception Laboratory, Department of Otolaryngology-Head and Neck Surgery, Western University, London, Ontario Canada

**Keywords:** 22q11.2 deletion, Non-syndromic cleft palate, Velopharyngeal dysfunction, Velopharyngeal insufficiency

## Abstract

**Objective:**

To identify and describe the dynamic features of velopharyngeal dysfunction (VPD) in patients with 22q11.2 deletion syndrome relative to patients with non-syndromic cleft palates.

**Study design:**

Retrospective case-control study.

**Setting:**

Pediatric tertiary care center.

**Subjects and methods:**

A total of 30 children (aged 9–16 years) with VPD were included in this study. Fifteen children with a definitive diagnosis of 22q11.2 deletion syndrome requiring surgical VPD repair were included in the 22q11.2 deletion syndrome group. Fifteen age- and sex-matched children with non-syndromic cleft palate requiring surgical VPD repair were included in the non-syndromic cleft palate group for comparison. Velar displacement, lateral pharyngeal wall displacement, and lateral pharyngeal wall motion pattern data were extracted from preoperative Multiview Videofluoroscopy imaging studies of all children and compared across groups.

**Results:**

Lateral pharyngeal wall displacement was found to be reduced in the 22q11.2 deletion syndrome group (U = 29.50, *p* = .001, r = .63). However, measures of velar displacement were not observed to differ between groups. Similarly, lateral pharyngeal wall motion pattern distributions were not found to differ across these two groups.

**Conclusions:**

Velopharyngeal dysfunction in patients with 22q11.2 deletion syndrome showed differences in dynamic velopharyngeal function when compared to non-syndromic cleft palate patients. The current findings provide initial insights into the unique aspects of velopharyngeal dysfunction for patients with 22q11.2 deletion syndrome. These findings may guide further efforts directed toward understanding the dynamic velopharyngeal characteristics of this population and potentially optimize surgical management and functional outcomes.

## Introduction

As a clinical diagnostic group, 22q11.2 deletion syndrome (22q11.2DS) is the most commonly identified human microdeletion syndrome with an estimated prevalence of 1 in 2000 to 7000 people [1–3]. Although it may be acquired via an autosomal dominant inheritance pattern, it most often occurs sporadically through a de novo mutation [4–6]. Before its genomic basis was explicitly defined, the eclectic clinical presentations of those with 22q11.2DS led to its description as a variety of clinical entities including DiGeorge syndrome, velocardiofacial syndrome, and conotruncal anomaly face syndrome. However, it is now known that its distinct multi-systemic clinical features (e.g., almond-shaped eyes, bulbous nasal tip, cardiac defects, immunodeficiency, developmental delays, psychiatric disorders, endocrine abnormalities, and palatal defects) are the result of the deletion of band q11.2 of chromosome 22 [7–12].

The majority of those identified with 22q11.2DS present with palatal defect-related velopharyngeal dysfunction (VPD) with a resultant range of abnormalities in speech production [12, 13]. Anatomical and physiological anomalies including tonsillar hypertrophy, adenoid hypoplasia, platybasia, hypotonicity, congenital velar shortening, and muscular abnormalities of the pharynx have been documented and hypothesized as factors contributing to the relatively high incidence of VPD among this specific population [1, 14–16]. Moreover, the aforementioned studies have noted that both structural and physiological deficits contribute to the presence of VPD in individuals with 22q11.2DS.

One consistently uniform pattern of VPD has not yet been documented. Thus, deficits related to VPD tend to vary significantly in both presentation and perceived severity, an observation that is likely to be the result of the variation in the velopharyngeal system and, subsequently, VPD. It is important to note, however, there is a paucity of literature regarding the range and complexity of the changes that underlie velopharyngeal abnormalities and their subsequent speech-related deficits specific to 22q11.2DS. Accordingly, the relatively rare nature of this diagnostic entity combined with its considerable VPD-related variability poses substantial clinical challenges in identifying and describing deficits specific to this population. Despite its rarity, the ability to gather descriptive information about 22q11.2DS may benefit both clinical classification and approaches to treatment.

Clinical information suggests that patients with VPD secondary to 22q11.DS are treated similarly to patients with non-syndromic cleft palate (NSCP) [17]. Regardless of etiology, treatment goals are directed towards improving velopharyngeal closure [17]. More specifically, these interventions seek to reduce the extent and impact of structural abnormalities on speech production by employing surgical methods, where indicated, that facilitate improved velopharyngeal closure during deglutition and phonation. Currently, there are several surgical approaches which have been shown to be effective interventions for VPD: posterior pharyngeal wall augmentation, Furlow palatoplasty, pharyngeal flap, sphincter pharyngoplasty, or combined Furlow palatoplasty and sphincter pharyngoplasty [17–21].

Despite similar surgical management, numerous studies have demonstrated that patients with 22q11.2DS have inferior post-operative speech outcomes and higher surgical revision rates when compared to patients with NSCP [22, 23]. The cause for these differences in clinical outcome has not yet been clearly identified but may emerge from variations in the dynamic restrictions underlying VPD in those with 22q11.2DS. Investigation of the anatomical factors and dynamic features of the velopharyngeal port in this specific patient population may, therefore, facilitate improved understanding of surgical considerations unique to those with 22q11.2DS. Although it is suspicioned that poorer speech outcomes in patients with 22q11.2DS are the result of multiple deficits that influence the velopharyngeal port, data to support this suggestion are currently limited. It is not unreasonable to anticipate that the dynamic function of the velopharyngeal port may be increasingly influenced as more structures within this complex region are disrupted; thus, speech deficits of 22q11.2DS may differ quantitatively from those that are more commonly noted in patients presenting with NSCP due to anatomical and functional differences inherent to 22q11.2DS specifically. Moreover, additional extensive functional disruptions in the presence of altered anatomy may provide more significant management challenges for this specific population. Information of this type would expand our knowledge base relative to not only the presence and severity of VPD in this population, but potentially the range of variability that might exist and how this may influence speech.

Thus, exploration of an array of anatomical factors in patients with 22q11.2DS-related VPD may facilitate both improved understanding of the static and functional changes unique to this specific population. These data may be of substantial clinical benefit from both a diagnostic and management perspective and assist in understanding the underlying causes of the discrepant postsurgical outcomes of patients with 22q11.2DS and NSCP undergoing VPD correction. Accordingly, the current study sought to investigate differences in dynamic anatomical features of VPD amongst patients with 22q11.2DS and NSCP. More specifically, we sought to investigate how velar displacement (VD), lateral pharyngeal wall displacement (LWD), and lateral pharyngeal wall motion pattern (LWMP) differ amongst these two distinct patient populations. We anticipate that these data will serve to enhance currently limited knowledge regarding the unique challenges faced by this rare clinical entity and provide insightful information which may be of clinical benefit from both a diagnostic and treatment perspective.

## Methods

### Patients

A retrospective case-control study was conducted by reviewing the medical records of patients who presented to the VPD clinic at the London Health Sciences Centre from 2001 to 2016. The study was approved by Western’s Research Ethics Board (100970). The inclusion criteria for the 22q11.2DS group consisted of 1) a definitive diagnosis of 22q11.2DS with fluorescence in situ hybridization, 2) the presence of VPD requiring surgical intervention, and 3) available preoperative Multiview Videofluoroscopy (MVV) imaging. Age- and sex-matched patients with NSCP were included for comparison. The inclusion criteria for the NSCP group consisted of 1) cleft palate, 2) genetic assessment found to have no genetic syndrome, 3) the presence of VPD requiring surgical intervention, and 4) availability of preoperative MVV imaging. No exclusion criteria were set regarding the presence of comorbidities or the type of cleft palate for either group.

### Acquisition of Velopharyngeal Measures

VD, LWD, and LWMP measures were extracted from the completed preoperative MVV imaging studies by a single clinician (CR). All measurements were obtained with a standard ruler from the frame with the greatest degree of displacement during consonant-vowel productions according to the standardized measurement procedures described by Golding-Kushner [24].

LWMP was defined as the lateral pharyngeal wall contour or shape during phonation [25–27]. As previously described, possible shapes for classification included shelf, balloon, vertical, and irregular [24]. VD was defined as the displacement of the velum along its anterior-posterior movement trajectory towards the posterior oropharyngeal wall. Lastly, LWD was defined as the maximal medial excursion of the lateral pharyngeal wall.

All displacements were recorded as a proportion of maximal theoretical displacement in each patient to standardize velar and pharyngeal measures across all patients. More specifically, a VD of 0 indicated no velar displacement towards the posterior oropharyngeal wall during phonation, while a value of 1 indicated complete anteroposterior velar closure (i.e., velar contact with the posterior oropharyngeal wall). Similarly, an LWD value of 0 indicated no transverse displacement of the lateral pharyngeal wall towards the opposite lateral wall of the pharynx; an LWD value of 1 represented the maximum theoretical LWD (i.e., complete medial excursion extending toward the opposite lateral pharyngeal wall); a LWD of 0.5 corresponded to the midline of the pharynx. In cases where the LWD was asymmetrical, the side with the greatest degree of displacement was identified.

The collective findings of past explorations confirm that variability in the structural shape and associated movement of the velopharyngeal port is to be expected [25–27]. With this information in mind, it may be reasoned that objective measurement of area and displacement lends itself ideally to use of this type of ratio index. Accordingly, data was obtained in such a standardized manner to permit direct comparison between populations without confounds associated with expected variability in velopharyngeal cross-sectional area [28, 29].

### Data analysis

Group comparisons of VD and LWD were conducted by two separate Mann-Whitney U tests. Between-group differences of LWMP distributions were assessed with a Fisher’s Exact Test. In an explicit effort to reduce interpretive errors, a Bonferroni correction was applied to control for alpha inflation related to multiple hypothesis testing. Thus, significance was assessed at α = 0.017. All statistical analyses were conducted using IBM SPSS statistics software package version 25 (IBM Corp., Armonk, N.Y., USA).

## Results

**Fig. 1:**
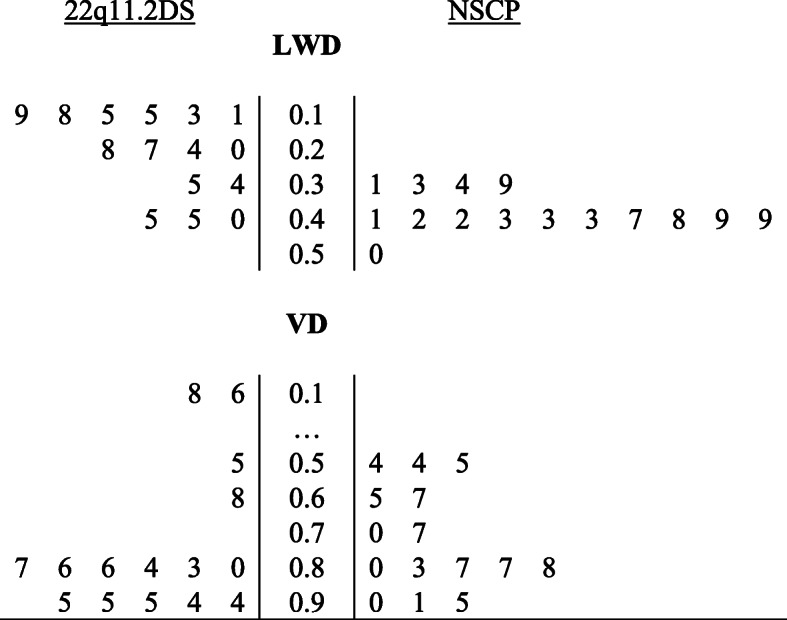
Distribution of lateral pharyngeal wall and velar displacement measures by group. 22q11.2DS: 22q11.2 Deletion Syndrome; LWD: lateral pharyngeal wall displacement; NSCP: non-syndromic cleft palate; VD: velar displacement

Sixteen patients with 22q11.2DS who underwent preoperative MVV imaging for surgical VPD correction were identified. One was excluded due to poor MVV quality related to patient age and cooperation, resulting in 15 (7 males; 8 females) patients. Fifteen age- and sex-matched controls with NSCP-related VPD who underwent preoperative MVV imaging studies were included for comparison. Thus, a total of 30 patient records were reviewed for the current study. A summary of patient characteristics stratified by group is presented in Table 1. Distributions of VD and LWD measures are shown in Fig. 1. The mean age of participants was 9.0 and 9.4 years for the 22q11.2DS and NSCP groups, respectively. Both groups ranged from 4 to 16 years of age. Most patients across both groups were observed to exhibit a balloon-type LWMP (*n* = 22); the remainder exhibited a shelf-type pattern (*n* = 8).

**Table 1:** Patient characteristics stratified by group

22q11.2DS						NSCP					
Patient	Age	Sex	LWD	LWMP	VD	Patient	Age	Sex	LWD	LWMP	VD
1	5	F	0.28	S	0.86	16	7	F	0.41	S	0.90
2	5	F	0.40	S	0.94	17	6	F	0.34	B	0.54
3	5	F	0.45	S	0.55	18	7	F	0.48	S	0.54
4	14	F	0.20	B	0.83	19	14	F	0.49	S	0.67
5	5	F	0.19	B	0.68	20	6	F	0.43	S	0.70
6	5	F	0.24	S	0.94	21	5	F	0.43	S	0.95
7	4	M	0.15	B	0.16	22	4	M	0.39	S	0.55
8	11	M	0.13	S	0.95	23	10	M	0.49	S	0.87
9	14	F	0.35	S	0.84	24	15	F	0.33	S	0.91
10	16	M	0.27	B	0.80	25	16	M	0.50	S	0.88
11	13	M	0.18	B	0.95	26	13	M	0.47	S	0.80
12	16	F	0.45	S	0.87	27	16	F	0.42	S	0.87
13	7	M	0.34	S	0.86	28	5	M	0.43	S	0.77
14	7	M	0.11	B	0.18	29	8	M	0.42	S	0.65
15	8	M	0.15	B	0.95	30	9	M	0.31	B	0.83
Range			0.11–0.45		0.16–0.95				0.33–0.50		0.54–0.95
Mean	9.0		0.26		0.76		9.4		0.42		0.76
SD	4.3		0.11		0.25		4.1		0.06		0.14
Median	7.0		0.24		0.86		8.0		0.43		0.80
IQR	8.5		0.18		0.20		7.5		0.08		0.22

*Note.* 22q11.2DS: 22q11.2 Deletion Syndrome; Shape of Pharyngeal Wall: Balloon (B), Shelf (S); LWD: lateral pharyngeal wall displacement; LWMP: lateral pharyngeal wall motion pattern; VD: velar displacement; NSCP: non-syndromic cleft palate; SD: standard deviation; IQR: Interquartile Range

Seven patients (46.7%) presented with a balloon-type LWMP and 8 (53.3%) showed a shelf-type LWMP in the 22q11.2DS group. In contrast, only two patients (13.3%) in the NSCP group showed a balloon-type LWMP; the remaining 13 NSCP patients (86.7%) showed a shelf-type LWMP. Although balloon-type LWMP appeared to be more common in patients with 22q11.2DS, between-group differences in distribution did not achieve statistical significance based on the Bonferroni-adjusted alpha of 0.017 (OR 12.25, *p = .*04). Similarly, there was no significant between-group difference in VD (0.86 vs. 0.80) (*U =* 93, *p =* .42, *r =* .15). In contrast, however, LWD was significantly more restricted in the 22q11.2DS group as compared to the NSCP group (0.24 vs. 0.43, respectively) (*U =* 29.50, *p* = .001, *r* = .63).

## Discussion

This study was conducted to identify and describe dynamic velopharyngeal function in patients with VPD associated with 22q11.2DS and NSCP. To aid in the interpretation of the results, data from age- and sex-matched NSCP patients requiring VPD correction were collected and used as a point of reference. We acknowledge that the use of those with NSCP-related VPD as a control group carries considerable restrictions; however, we intentionally chose this group for a basis of comparison in an attempt to better understand the nature of VPD and the potential dynamic differences underlying the discrepant postsurgical outcomes between 22q11.2DS and NSCP VPD repair. Thus, these two clinical groups were assessed with an a priori assumption that patients with NSCP-related VPD could provide a “point of reference” from which unique patterns of variability might be detected.

Based on our data, VPD patients with 22q11.2DS exhibited significant differences in dynamic velopharyngeal function as seen on MVV compared to those with NSCP. However, the significance should not be interpreted to indicate that two distinct groups exist; rather, we believe the current data support the assumption that those with 22q11.2DS may exhibit variation in VPD relative to those with NSCP.

A salient finding from our study is that those with 22q11.2DS exhibited LWDs that were reduced by almost half compared to that identified for the VPD control group (0.24 vs. 0.43).

Inadequate velopharyngeal closure results in velopharyngeal incompetence which can manifest as speech hypernasality, decreased speech intelligibility, and inappropriate nasal emissions. It is well-documented that variability in the broader diagnostic category of “VPD” is substantial [25–27]. However, we also believe that the ability to describe and further develop datasets on particular subgroups of those with VPD is essential in order to better understand the deficits. From the standpoint of speech production, minor changes in structure and function of this dynamic region may create substantial reductions in the effectiveness of not only VP closure, but the end-product of speech that is produced. It is our opinion that if the extent of deficits can be quantified, more exact prospective studies on the evolution of the problem, as well as the ability to monitor post-treatment outcomes will be enhanced.

It is important to note, however, that the external validity of the present data must be viewed with caution. This comment is raised due to two specific issues. First, in the present work, those with VPD secondary to NSCP were selected and used as a point of reference in the current descriptive exploration. Secondly, the sample size used is indeed small and with that comes the potential for differences to emerge with larger participant samples. However, we feel that the ability to carefully describe metrics in relatively rare populations allows for the generation of more specific questions about VPD in these populations. These data support the notion that LWD measures can serve to distinguish distinct subpopulations with abnormalities of the velopharyngeal system. Moreover, they provide a plausible rationale for the observed differences in the functional postoperative outcomes of those with 22q11.2DS and other groups with VPD. Hence, the present data provide a valuable, initial index of how this relatively rare syndrome compares to others with a similar deficit in VP functioning.

The current study also demonstrated a lack of velar displacement differences between the groups assessed. To fully appreciate this result, it should be noted that the 22q11.2DS patients were compared to those with cleft palate, rather than those secondary to a syndrome. Since NSCP patients would be expected to have lower than normal VD measures, we can extrapolate with some level of confidence that the 22q11.2DS patients would also have diminished VD than that which would be expected from the normal population. The combination of decreased LWD and less-than-expected VD may, at least in part, contribute to lowered success rates following surgery. Yet we must clearly acknowledge that additional data from a larger number of participants is necessary.

Treatment of VPD in patients with 22q11.2DS presents a unique challenge. Widdershoven et al. [23] reported a 16% revision rate in the 22q11.2DS compared to 0% in patients without, with the latter group also showing better outcomes on endoscopy and auditory-perceptual assessment of speech postoperatively. The patients examined by Widdershoven et al. all had initial palatal lengthening procedure whereas the revision was performed using sphincter pharyngoplasty. Similarly, Losken et al. [22] described their revision rate in 22q11.2DS patients to be significantly higher than in those without (22% vs 11%). For Losken et al., all of the patients studied underwent primary sphincter pharyngoplasty. The authors noted that patients who required revision of pharyngoplasty were more likely to have larger velopharyngeal areas. The higher revision rates, therefore, invite the search for intrinsic patient characteristics in patients with 22q11.2DS that may impact their postoperative outcome. Extrapolating from our results, it is possible that the decreased lateral wall movement hinders the patient’s ability to close the ports following a pharyngeal flap. The lack of dynamic closure in turn leads to unsatisfactory postoperative results. Hence, 22q11.2DS patients may benefit from a wider pharyngeal flap to compensate for the decreased dynamic lateral wall movement. Conversely, sphincter pharyngoplasty relies less on the existing lateral wall movement; instead, it narrows the velopharynx and may compliment the dynamic limitations seen in 22q11.2DS patients. The results of this study should be interpreted as to highlight dynamic differences in the 22q11.2DS patients from which surgeons can make adjustments in their technique in hopes of decreasing the revision rate.

In addition to the above findings, the pattern or type of velopharyngeal change during phonation is of interest. That is, both balloon- and shelf-type closure patterns were observed. However, a unique finding relative to these closure patterns is that those with 22q11.2DS were evenly divided, with 7 participants exhibiting a balloon-type and 8 exhibiting shelf-type closure. In contrast, within the NSCP reference group, 13 of the 15 patients were identified to have a shelf-type closure. Based on our review of the literature, we were unable to identify previous reports of this type of closure pattern in 22q11.2DS patients. This finding raises questions about unique structural differences that distinguish these two patient groups. For example, it is clear that anatomical differences will exist in association with this syndrome; however, the physiologic changes that emerge are likely to be quite variable. Additionally, the potential impact of volitional, function compensation(s) add a further dimension to this important concern. For that reason, additional exploration of this unique clinical population and the resultant variations that exist are warranted.

Given the structural complexities of this anatomical region, it is not unreasonable to suggest that minor variations in structure may result in subtle dynamic alterations that may be overlaid with active compensatory changes. At present, we are not sure of the underlying mechanism of the balloon-type closure pattern, nor can we comment on its clinical significance. It does, however, appear to be quantifiable when evaluating the individual data presented in Table 1, as well as when considering those data relative to the NSCP reference group.

When the present body of data is evaluated collectively, we anticipate additional explorations of this phenomenon would offer an interesting and valuable area for future study specific to both the visualization and measurement of the system and its impact on speech production. Defects within the velopharyngeal system will have real and direct influences on the acoustic structure of speech [30]. Consequently, detailed analyses of the acoustic properties of speech in those with 22q11.2DS may offer valuable insights into how changes similar to those we have documented influence intelligibility [31].

Although we believe the present study has provided valuable insights into the unique characteristics of patients with 22q11.2DS, several limitations deserve explicit mention. First, it is important to note that our results are limited by the relatively small number of patients. This reflects the phenotypic variability of the disease; that is, not all 22q11.2DS patients present with VPD that is severe enough to warrant referral to a tertiary VPD clinic. Furthermore, MVV is not universally used for patients with VPD since the diagnosis can also be achieved through nasopharyngoscopy [32]. We acknowledge that MVV and nasopharyngoscopy each present distinct advantages and disadvantages over each other. However, MVV was specifically chosen for this study because of its ability to more objectively quantify velopharyngeal measures in contrast to nasopharyngoscopy’s inherently more subjective interpretative evaluation which makes quantification of measurements much more difficult. Nevertheless, we wholeheartedly agree that clinicians should take advantage of both modalities of assessment as they provide crucial complimentary information required for adequate surgical management of VPD. Although our study lacks the presence of healthy controls, the comparison to those diagnosed with NSCP-related VPD provides valuable insight into the velopharyngeal characteristics that may contribute to the observed differences in postsurgical outcomes exhibited between these two groups. Despite the lack of exclusions regarding the presence of comorbidities or type of cleft palate, we feel that our study’s external validity is preserved as clinicians would regularly encounter similar challenges with heterogenous non-syndromic and 22q11.2DS cohorts. Nevertheless, this study is limited by the same factors that challenge many efforts which seek to gather information on clinical populations that are small in the context of otolaryngology. In this regard, findings must always be evaluated and interpreted with care; findings on limited albeit unique clinical populations have restriction specific to external validity. However, data such as ours suggest a starting point from which additional work can be replicated and hopefully verified.

Our study objectively quantifies dynamic velopharyngeal features in 22q11.2DS patients with VPD and improves our understanding of the distinct velopharyngeal features and deficiencies of this population. Given that different surgical approaches for VPD correction affect static and dynamic velopharyngeal function uniquely, one may consider complementing the unique velopharyngeal deficits of 22q11.2DS with the advantages of a specific surgical technique; however, further research investigating VPD surgical techniques and posttreatment outcomes in patients diagnosed with 22q11.2DS is required. Finally, additional details on speech performance and its consistency within across participants is necessary in future work.

## Conclusion

The current study has provided objective data on dynamic features of VPD in those with 22q11.2DS. Our findings revealed that patterns of VPD differ between those with and without 22q11.2DS. In particular, the LWD was found to be significantly reduced in patients with 22q11.2DS when data obtained from those with VPD secondary to NSCP served as a point of reference. These data provide valuable initial insights into the identification and description of the dynamic velopharyngeal function and its variability in association with 22q11.2DS-related VPD, as well as differences potentially underlying discrepant postsurgical outcomes. However, due to our relatively small sample size, the interpretation and extension of these data must be done with care. For that reason, additional investigation into the velopharyngeal characteristics of patients with 22q11.2DS may permit a more comprehensive understanding of the structural and functional deficits that influence postsurgical and speech outcomes and may serve surgeons in identifying optimal surgical management for this unique population.

## Abbreviations

VPD: Velopharyngeal dysfunction
22q11.2DS: 22q11.2 deletion syndrome
NSCP: Non-syndromic cleft palate
MVV: Multiview Videofluoroscopy
VD: Velar displacement
LWD: Lateral pharyngeal wall displacement
LWMP: Lateral pharyngeal wall motion pattern
